# Mining co-occurrence and sequence patterns from cancer diagnoses in New York State

**DOI:** 10.1371/journal.pone.0194407

**Published:** 2018-04-26

**Authors:** Yu Wang, Wei Hou, Fusheng Wang

**Affiliations:** 1 Department of Computer Science, Stony Brook University, Stony Brook, New York, United States of America; 2 Department of Family, Population and Preventive Medicine, Stony Brook University, Stony Brook, New York, United States of America; 3 Department of Biomedical Informatics, Stony Brook University, Stony Brook, New York, United States of America; Novo Nordisk Foundation Center for Protein Research, University of Copenhagen, DENMARK

## Abstract

The goal of this study is to discover disease co-occurrence and sequence patterns from large scale cancer diagnosis histories in New York State. In particular, we want to identify disparities among different patient groups. Our study will provide essential knowledge for clinical researchers to further investigate comorbidities and disease progression for improving the management of multiple diseases. We used inpatient discharge and outpatient visit records from the New York State Statewide Planning and Research Cooperative System (SPARCS) from 2011-2015. We grouped each patient’s visit history to generate diagnosis sequences for seven most popular cancer types. We performed frequent disease co-occurrence mining using the Apriori algorithm, and frequent disease sequence patterns discovery using the cSPADE algorithm. Different types of cancer demonstrated distinct patterns. Disparities of both disease co-occurrence and sequence patterns were observed from patients within different age groups. There were also considerable disparities in disease co-occurrence patterns with respect to different claim types (i.e., inpatient, outpatient, emergency department and ambulatory surgery). Disparities regarding genders were mostly found where the cancer types were gender specific. Supports of most patterns were usually higher for males than for females. Compared with secondary diagnosis codes, primary diagnosis codes can convey more stable results. Two disease sequences consisting of the same diagnoses but in different orders were usually with different supports. Our results suggest that the methods adopted can generate potentially interesting and clinically meaningful disease co-occurrence and sequence patterns, and identify disparities among various patient groups. These patterns could imply comorbidities and disease progressions.

## Introduction

### Background and significance

Patient level longitudinal data mining and pattern discovery is a common approach for public health studies. For example, disease co-occurrence patterns and disease sequence patterns from large number of patients’ diagnosis histories could help to discover comorbidity or disease progression patterns. Generally, disease co-occurrence patterns could imply comorbidities and disease sequence patterns could reveal disease progression. For instance, novel associations or generalized associations from raw data helped explore co-occurrence patterns of multiple diseases [1, 2]. Event sequences were mapped to a general knowledge representation model for mining longitudinal event data [3]. Specific techniques like windowing, episode rules and inductive logic programming were applied to identify sequential or temporal patterns related to cardiovascular diseases [4]. Sequential patterns were also helpful in analyzing temporal trends of diseases in certain situations [5]. However, due to the limitation of data availability, most previous studies were based on small datasets or a limited number of healthcare facilities.

Recently, open data initiatives from governments make available large amounts of healthcare data and provide researchers with a unique opportunity to study disease co-occurrence and sequence patterns. For example, an early project worked on discovering disease progression and mitigating potential adverse outcomes of chronic obstructive pulmonary disease (COPD) [6]. Another work used machine learning techniques such as hidden Markov models to study disparities among different cohorts by clustering patients into different groups using patient claim data [7]. Healthcare data were also combined with multiple data sources, such as social economic data and social media data, to predict hospital visits regarding certain diseases in specific areas [8].

As a representative of New York State’s open data initiative, the Statewide Planning and Research Cooperative System (SPARCS) [9] has been collecting patient level demographic and diagnosis information on discharges for over 35 years. All article 28 licensed facilities (i.e. hospitals, nursing homes, and diagnostic treatment centers) in New York State are required to report outpatient, inpatient, emergency department and ambulatory surgery discharge records to SPARCS every year [10]. It has already been used in big data analysis in medical domain, like evaluating the quality of data reporting [11] and discovering associations between various factors and outcomes of treatments [12–14]. Besides, SPARCS provides rich data for exploring spatial, temporal and spatio-temporal patterns of various diagnoses [15–18].

### Objective

This is a retrospective cohort study aiming at analyzing frequent disease co-occurrence and sequence patterns of cancer diagnoses in New York State. We studied disparities among these frequent patterns with respect to age, gender and claim types (i.e., inpatient, outpatient, emergency department and ambulatory surgery) for hospital visits or stays. Since cancer ranks the second in the leading causes of deaths in the United States [19], we believe that the results will provide essential data and knowledge for clinical researchers to further investigate comorbidities and disease progression for improving the management of cancers.

## Materials and methods

This study has been approved by Stony Brook University IRB (CORIHS B). We took advantage of the cancer-related diagnosis information available in SPARCS data, i.e., the ninth and tenth revision of International Classification of Diseases (ICD-9 and ICD-10) diagnosis codes, and converted them to single-level Clinical Classifications Software (CCS) diagnosis categories [20] to discover disease co-occurrence and sequence patterns from patients’ full diagnosis histories within a five-year time frame.

### Data sources

While our SPARCS data from most claim types are available as early as the year 2003, outpatient records are only available since 2011. To provide a comprehensive history of patient visits, we choose discharge records in SPARCS during 2011-2015 where all claim types are available. Descriptive statistics of cancer patients based on discharge records in SPARCS during 2011-2015 are presented in Table 1. Each discharge record contains one or more ICD-9 or ICD-10 diagnosis codes. The first diagnosis code is the primary diagnosis code that represents the main reason for that hospital visit. The rest are secondary diagnosis codes that represent the conditions coexisting during the same hospital stay or visit. To reduce dimensionality of data, we mapped ICD diagnosis codes to single-level CCS diagnosis categories and used CCS categories in our analyses. In this paper, both CCS diagnosis category descriptions and labels are used to represent various diagnoses. Since procedure codes were only available in a very small portion of records, we kept using ICD-9 or ICD-10 procedure codes without mapping. This study focuses on discovering patterns from seven types of cancer with high incident rates in New York State: rectum and anus cancer (15), liver and intrahepatic bile duct cancer (16), pancreas cancer (17), lung and bronchus cancer (19), breast cancer (24), prostate cancer (29) and Non-Hodgkin’s lymphoma (38) [21]. There are 8,645,995 discharge records from 742,487 patients used in our work.

**Table 1:** Patient characteristics of seven types of cancer in SPARCS, 2011-2015.

Patient characteristics	Cancer						
	Lung and bronchus	Rectum and anus	Pancreas	Liver*	Non-Hodgkin’s lymphoma	Prostate	Breast
**Total population, n**	120,833	40,816	25,352	28,190	75,718	197,847	300,682
**Age, years**							
Mean (std)	68.37 (12.16)	63.07 (14.70)	67.72 (13.14)	63.37 (13.89)	61.81 (18.05)	71.27 (10.90)	64.26 (14.49)
Median (min, max)	69 (0, 111)	63 (0, 102)	68 (0, 121)	64 (0, 106)	64 (0, 112)	71 (0, 111)	64 (0, 124)
<= 34, n (%)	1,071 (0.89)	1,367 (3.35)	305 (1.20)	828 (2.94)	6,699 (8.85)	279 (0.14)	4,993 (1.66)
35-54, n (%)	14,397 (11.91)	10,146 (24.86)	3,537 (13.95)	5,251 (18.63)	15,681 (20.71)	12,148 (6.14)	76,410 (25.41)
55-74, n (%)	65,479 (54.19)	19,643 (48.13)	13,313 (52.51)	16,177 (57.39)	33,440 (44.16)	106,400 (53.78)	140,758 (46.81)
>= 75, n (%)	39,886 (33.01)	9,660 (23.67)	8,197 (32.33)	5,934 (21.05)	19,898 (26.28)	79,020 (39.94)	78,521 (26.11)
**Gender**							
Male, n (%)	58,179 (48.15)	20,791 (50.94)	12,669 (49.97)	17,311 (61.41)	39,031 (51.55)	197,847 (100.00)	3,914 (1.30)
Female, n (%)	62,651 (51.85)	20,021 (49.05)	12,682 (50.02)	10,879 (38.59)	36,684 (48.45)	0 (0.00)	296,762 (98.70)
**Race**							
White, n (%)	88,497 (73.24)	27,316 (66.92)	17,054 (67.27)	16,201 (57.47)	54,159 (71.53)	133,385 (67.42)	207,472 (69.00)
Black or African American, n (%)	12,454 (10.31)	5,014 (12.28)	3,357 (13.24)	3,972 (14.09)	6,938 (9.16)	31,067 (15.70)	34,199 (11.37)
Native American or Alaskan Native, n (%)	236 (0.20)	110 (0.27)	40 (0.16)	78 (0.28)	153 (0.20)	415 (0.21)	607 (0.20)
Asian, n (%)	3,707 (3.07)	1,426 (3.49)	801 (3.16)	2,056 (7.29)	1,643 (2.17)	2,994 (1.51)	8,478 (2.82)
Native Hawaiian or Other Pacific Islander, n (%)	225 (0.19)	65 (0.16)	35 (0.14)	50 (0.18)	101 (0.13)	375 (0.19)	531 (0.18)
Other Race, n (%)	13,926 (11.52)	6,199 (15.19)	3,704 (14.61)	5,430 (19.26)	11,349 (14.99)	26,504 (13.40)	43,359 (14.42)
**Ethnicity**							
Spanish/Hispanic Origin, n (%)	7,154 (5.92)	3,533 (8.66)	1,956 (7.72)	3,257 (11.55)	6,002 (7.93)	14,267 (7.21)	22,173 (7.37)
Not of Spanish/Hispanic Origin, n (%)	108,549 (89.83)	35,403 (86.74)	22,403 (88.37)	23,841 (84.57)	66,437 (87.74)	174,834 (88.37)	264,583 (87.99)

* Liver includes intrahepatic bile duct.

### Data preparation

Each patient’s discharge records were grouped together using an encrypted unique patient identifier in SPARCS and ordered by the corresponding admission dates. Discharge records containing AIDS/HIV or abortion diagnoses were removed from our analyses, because their admission dates and patient identifiers were redacted to comply with the Health Insurance Portability and Accountability Act (HIPAA) [22]. Patient level demographic information (i.e. age, gender, race and ethnicity) were collected from the first record of each patient. Patients were classified into cohorts having different types of cancer. A patient who had any cancer diagnosis was selected into the corresponding cohort. One patient could be in multiple cohorts because a person could have been diagnosed with different types of cancer. Table 1 shows the patient characteristics in our analyses. For each type of cancer, we studied the disparities of top 20 frequent co-occurrence and sequence patterns among different age groups (<= 34, 35-54, 55-74 and >= 75 years old) [23] and gender groups (male and female). We also analyzed disparities of co-occurrence patterns using discharge records from different claim types. Disparities among different race and ethnicity groups are not discussed in this paper, but we provide relevant results generated using discharge records from patients who had Non-Hodgkin’s lymphoma (38) in S7 Table.

### Patients’ diagnosis sequences

**Fig 1:**
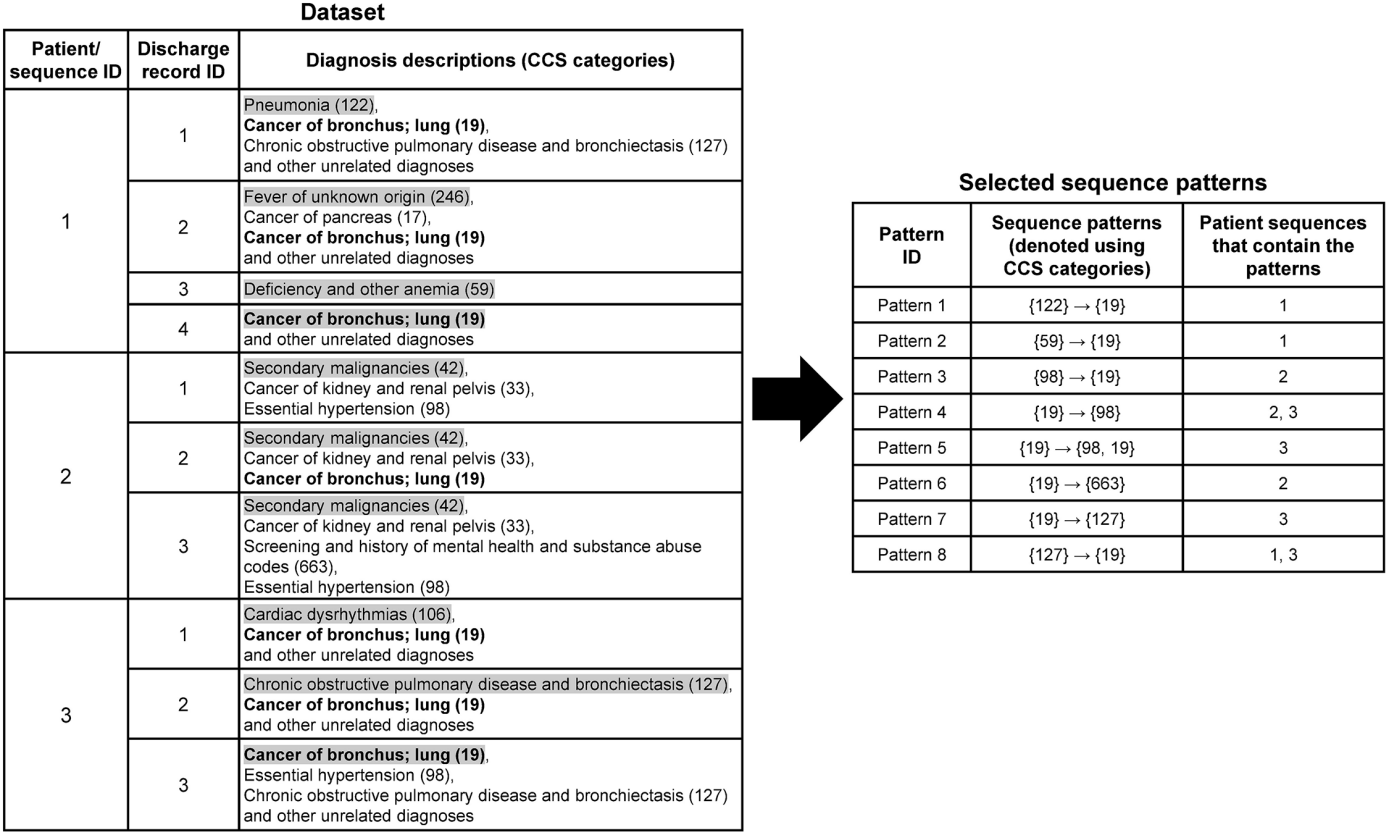
A random example of diagnosis sequences from patients having lung and bronchus cancer (19).

For each patient, since discharge records were strictly ordered by admission dates, diagnosis information was also strictly ordered by corresponding admission dates. Thus, each patient’s admission dates and diagnosis information constituted a diagnosis sequence. Each patient’s diagnosis sequence was assigned a unique sequence ID, which was also an ID for this patient. Fig 1 shows a randomly selected example consisting of three diagnosis sequences from patients having lung and bronchus cancer (19), which is the targeted cancer in this example. Diagnoses are listed after the discharge record ID, a corresponding CCS category is marked in the parentheses following the description. The primary diagnosis in each discharge record is emphasized using grey shading. CCS category that represents the targeted cancer (i.e., lung and bronchus cancer) is highlighted in bold. In this example, pattern 5 (“{19}→{98, 19}”) means that diagnosis “{cancer of bronchus; lung (19)}” happens before diagnoses “{essential hypertension (98), cancer of bronchus; lung (19)}”. The former one diagnosis and the latter two diagnoses occur in different discharge records on different admission dates. And the latter two diagnoses occur on the same day.

### Analysis methods

#### Apriori algorithm: Identifying frequent disease co-occurrence patterns

We adopted the Apriori algorithm [24] to discover frequent disease co-occurrence patterns and frequent procedure codes. The Apriori algorithm works by making multiple passes over the entire dataset and generating frequent co-occurrence itemsets (i.e., CCS categories on the same admission date) by comparing their supports with a user-specified minimum support threshold. If the minimum support threshold is satisfied, this co-occurrence itemset is kept in the searching results; otherwise it is deleted from the searching results. In the first pass, the algorithm simply counts occurrences (i.e., support) of each CCS category and procedure code and determines which of them are large (i.e., satisfy the minimum support threshold). In each subsequent pass, there are two phases. First, the algorithm starts with large itemsets found in the previous pass to generate new potentially large itemsets, say candidate itemsets, by joining new CCS categories or procedure codes. Next, the dataset is scanned to calculate the support of each candidate itemset, and determine large itemsets in the current pass by comparing these supports with the minimum support threshold. Eventually, all co-occurrence itemsets containing disease codes or procedure codes and satisfy the minimum support threshold are generated.

Both primary and secondary CCS categories were used in the analysis of disease co-occurrence patterns. Only records containing targeted cancer CCS categories were selected. For instance, in the sequences illustrated in Fig 1, discharge records containing “Cancer of bronchus; lung (19)” were used, and diagnoses in the same discharge record would be included in a large itemset if they satisfied the minimum support. We discovered co-occurrence relationships not only between different diagnoses, but also between diagnoses and procedures. As for associations between cancers and procedures, since not all records contained valid procedure codes, we only chose records containing both targeted cancer CCS categories and valid procedure codes in this analyses. For each type of cancer, we selected the top 20 potentially meaningful co-occurrence itemsets that contained targeted cancer diagnoses as frequent co-occurrence patterns. We used apyori 1.1.1 [25], a Python package for Apriori algorithm to discover frequent co-occurrence patterns.

#### cSPADE algorithm: Discovering frequent disease sequence patterns

We used cSPADE, a frequent sequence mining algorithm [26] to discover frequent disease sequence patterns for different types of cancer. cSPADE algorithm generates frequent sequences iteratively based on a subsequence relation that if a sequence is frequent, then all subsequences of this sequence are also frequent [27]. In each iteration, the cSPADE algorithm also works by comparing the supports of candidate sequences with the minimum support threshold. It starts from the single item sequence, to sequences with maximal length by joining subsequences obtained from previous iteration. When computing the support of a subsequence, multiple occurrences of this subsequence in the same sequence are counted only once.

Fig 1 shows an example of diagnosis sequences containing both primary and secondary CCS categories where primary CCS categories are in grey shading. The length of a sequence pattern is the total number of itemsets in this sequence. For example, pattern 5, which means “{19}” happens before “{98, 19}”, is a length-2 sequence pattern because there are two itemsets “{19}” and “{98, 19}” in this sequence. We set the minimum interval between two itemsets as one and the maximum interval as 180, such that the duration between admission dates of two consecutive itemsets in a sequence pattern must be within 1-180 days. That is, this algorithm can discover the association of two diagnoses happen within 180 days of each other. Previous studies found out that revisit intervals usually range from one month to over one year, and typical intervals are two, three or six months [28]. Thus, 180 days is an interval long enough to cover significant revisit diagnoses. For each type of cancer, we also kept the first 20 potentially meaningful subsequences that contained targeted cancer diagnoses as frequent sequence patterns. All frequent sequence patterns were mined using arulesSequences [29], a R package for cSPADE algorithm.

#### Statistical analyses

We selected top 20 co-occurrence patterns and top 20 sequence patterns to run statistical analyses for each type of cancer. Percentages of co-occurrence and sequence patterns were calculated and compared between age, gender, race, ethnicity group and claim type to evaluate disparities among these patient groups. For each of top co-occurrences as the dichotomous outcome, a generalized linear mixed-effect model was fit for the repeated measure data. Age, gender, race, ethnicity and claim type were all included in the same model as covariates. The within-subject dependence over repeated visits was adjusted using an unstructured covariance matrix. *p*-values based on *F*-tests were assessed to evaluate the overall significance of those covariates. For sequence patterns, multiple logistic regression models were fit for each sequence pattern as the dichotomous outcome. Similar to the analyses of co-occurrences, age, gender, race and ethnicity were treated as covariates and *p*-values based on *F*-tests were used to assess their overall significance. The Bonferroni’s method was used to adjust *p*-values for multiple tests. All statistical analyses were performed using SAS v9.4 (the SAS Institute, Cary, NC) [30].

## Results

We performed analyses on patients’ diagnosis histories and focused on seven types of cancer with high incident rates in New York State. Meaningless results, such as patterns consisting of identical CCS categories, CCS categories that represent unspecific disease groups or serve administrative purposes, length-1 patterns and patterns irrelevant to targeted cancers, were ruled out from our analyses.

In this section, we mainly discuss patterns containing diagnoses closely related to the targeted cancers and present results of a few cancer types where significant pattern disparities are found. Other results are available in the supporting information.

### Diagnosis co-occurrence patterns

We analyzed disparities of co-occurrence patterns among different age groups, gender groups and records from different claim types. Besides, we also discovered correlations between cancer diagnoses and procedures to see what procedures were frequently adopted to treat different types of cancer.

#### Frequent disease co-occurrence patterns in different age groups

**Fig 2:**
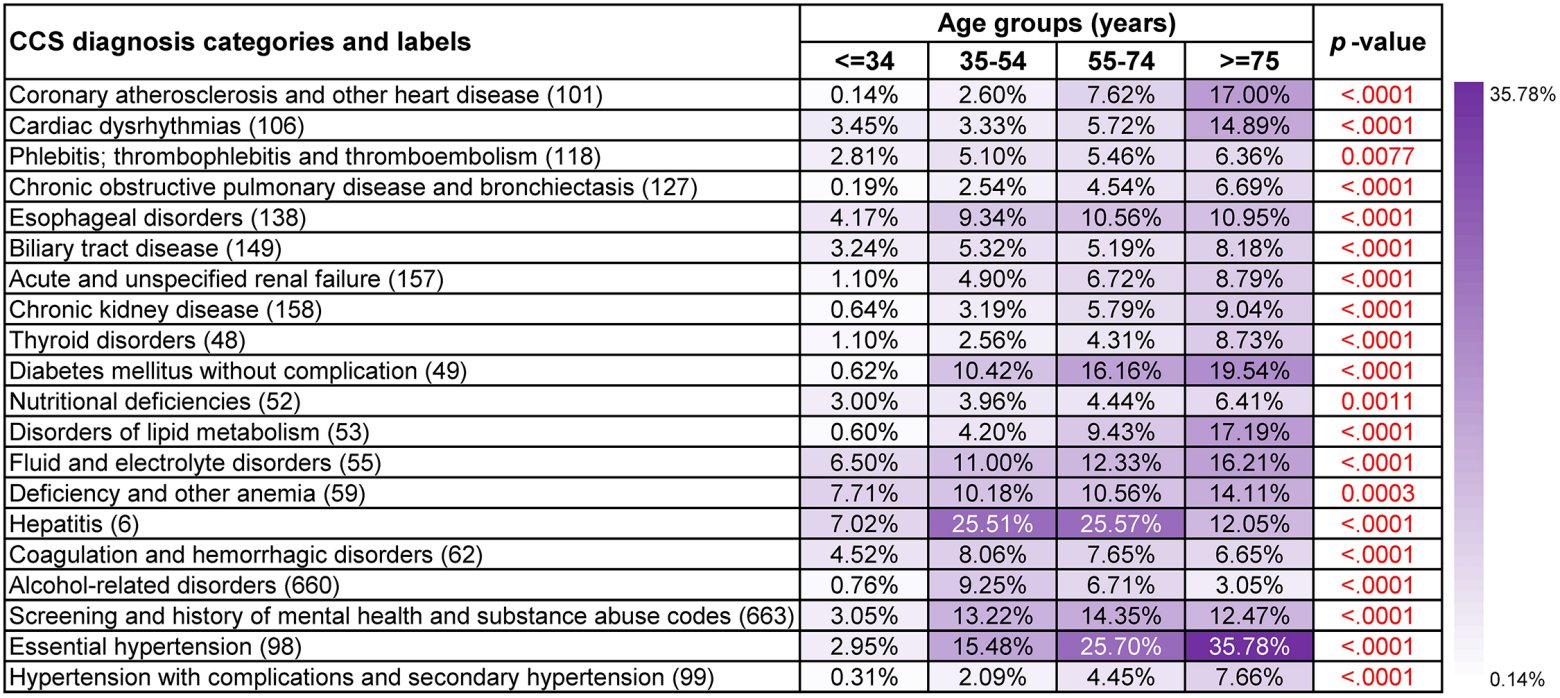
Supports and *p*-values of the most frequent diagnoses that co-occurred with liver and intrahepatic bile duct cancer (16) in different age groups. Age, gender, race, ethnicity and claim type were treated as covariates and *p*-values based on *F*-tests were used to assess their overall significance.

**Fig 3:**
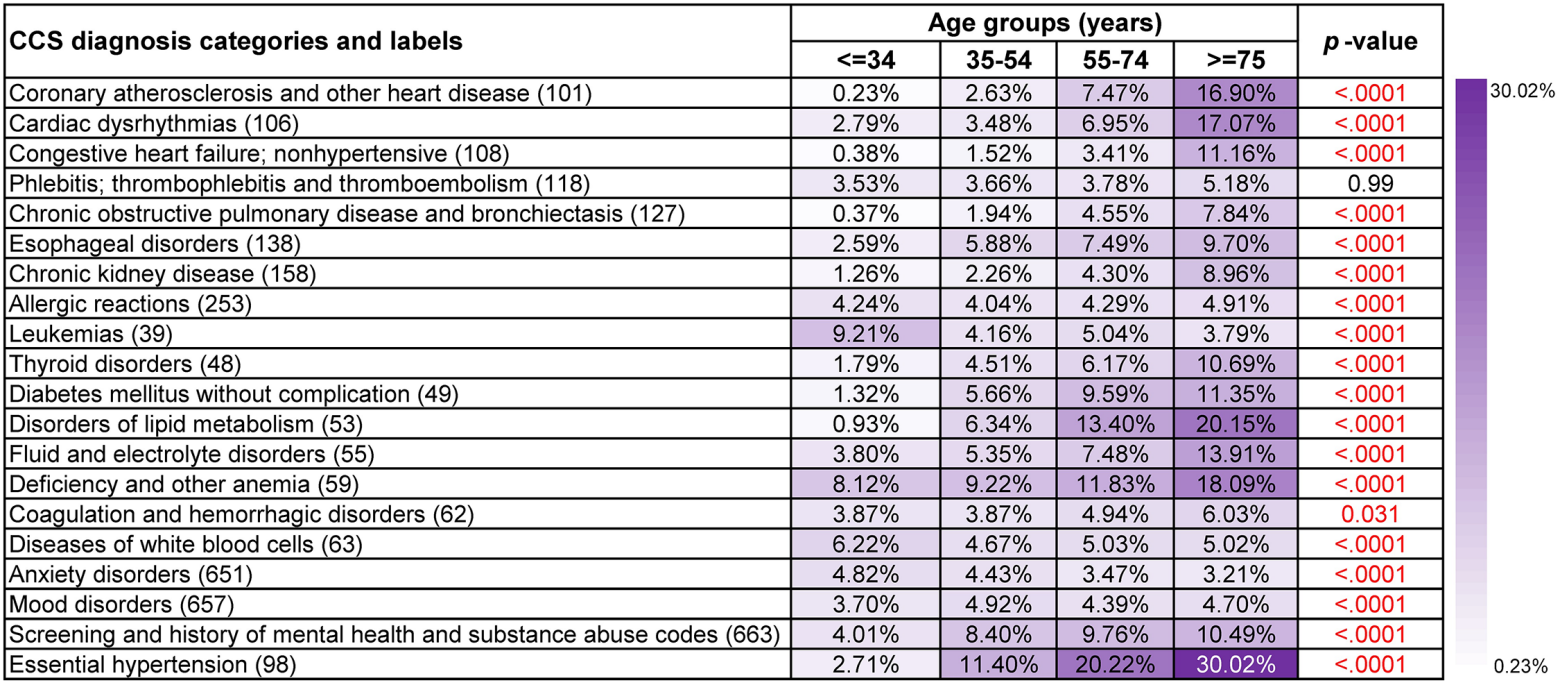
Supports and *p*-values of the most frequent diagnoses that co-occurred with Non-Hodgkin’s lymphoma (38) in different age groups. Age, gender, race, ethnicity and claim type were treated as covariates and *p*-values based on *F*-tests were used to assess their overall significance.

Figs 2 and 3 show supports and *p*-values of top 20 frequent diagnoses that co-occurred with liver and intrahepatic bile duct cancer (16) and Non-Hodgkin’s lymphoma (38), respectively. The *p*-values revealed that almost all of these diagnoses were significant with respect to age. Besides, supports of many top frequent diagnoses were the highest in the eldest age group (>= 75 years old) and were the lowest in the youngest age group (<= 34 years old).

Fig 2 presents results of frequent co-occurrence patterns regarding liver and intrahepatic bile duct cancer (16). Among patients who were or under 34 years old, deficiency and other anemia (59) and hepatitis (6) were two most popular diagnosis co-occurrences (7.71% and 7.02%, respectively). Patients between 35-74 years old were also more likely to have hepatitis (6), while it was less seen among patients who were or over 75 years old (35-54 years old: 25.51%, 55-74 years old: 25.57%, >= 75 years old: 12.05%, *p*-value<0.0001). Essential hypertension (98) was another diagnosis co-occurrence that usually occurred among patients who were or over 55 years old (55-74 years old: 25.70%, >= 75 years old: 35.78%, *p*-value<0.0001). Results for Non-Hodgkin’s lymphoma (38) were usually more representative and less noisy (Fig 3). For instance, leukemias (39) and diseases of white blood cells (63) were more frequent among patients who were or under 34 years old (9.21% and 6.22%, respectively). Essential hypertension (98) was also the most popular diagnosis co-occurrence with patients who were or over 55 years old (55-74 years old: 20.22%, >= 75 years old: 30.02%).

Patients who had rectum and anus cancer (15) (S1 Table) were more frequently diagnosed with cancer of colon (14). However, this diagnosis was not significant with respect to age (*p*-value = 0.99). Biliary tract disease (149) and diabetes mellitus without complication (49) were significant diagnoses (*p*-value<0.0001) that co-occurred with pancreas cancer (17) (S3 Table). As for lung and bronchus cancer (19) (S4 Table), the frequent diagnosis most relevant to this cancer was chronic obstructive pulmonary disease and bronchiectasis (127), which ranked high on the list of the most frequent co-occurrence patterns for patients who were or over 55 years old (55-74 years old: 19.30%, >= 75 years old: 26.01%, *p*-value<0.0001). Pneumonia (122) was another frequent diagnosis that was significant with respect to age (*p*-value<0.0001). Nonmalignant breast conditions (167) co-occurred more frequently with breast cancer (24) (S5 Table) among people who were or under 54 years old (<= 34 years old: 7.06%, 35-54 years old: 8.40%). For co-occurrence patterns among patients who had prostate cancer (29) (S6 Table), genitourinary symptoms and ill-defined conditions (163) was a significant sign of patients who were or under 34 years old (12.27%).

#### Frequent disease co-occurrence patterns in different gender groups

**Fig 4:**
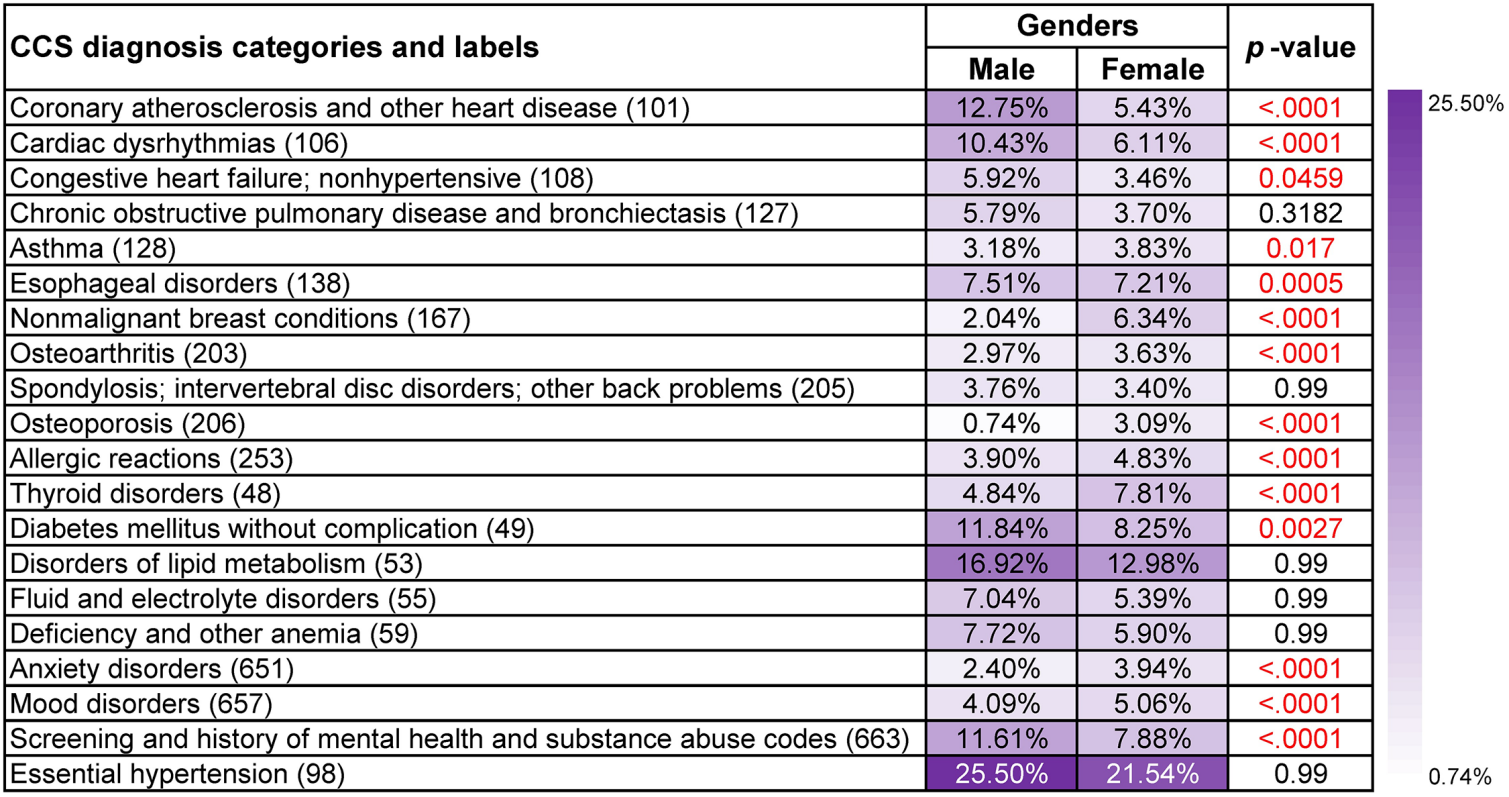
Supports and *p*-values of the most frequent diagnoses that co-occurred with breast cancer (24) in different gender groups. Age, gender, race, ethnicity and claim type were treated as covariates and *p*-values based on *F*-tests were used to assess their overall significance.

The most frequent diagnosis co-occurrences for cancer types that are less gender specific, such as rectum and anus cancer (15) (S1 Table), liver and intrahepatic bile duct cancer (16) (S2 Table), pancreas cancer (17) (S3 Table) and lung and bronchus cancer (19) (S4 Table), demonstrated similar trends in males and females with only very few disparities. For example, liver and intrahepatic cancer (16), females were usually at a higher risk of having deficiency and other anemia (59) than males (male: 10.32%, female: 12.14%, *p*-value<0.0001). Males were more likely to have hepatitis (6) compared with females (male: 25.69%, female: 16.83%, *p*-value<0.0001). Thyroid disorders (48) was more popular with females among patients who had Non-Hodgkin’s lymphoma (38) (S7 Table) and breast cancer (24) (Fig 4), but was not high on the list of the top frequent disease co-occurrences for males (*p*-values<0.0001). It can also be observed that heart disease like coronary atherosclerosis (101) affected males more than females across all seven types of cancer (*p*-values<0.0001).

#### Frequent co-occurrence patterns from different claim types

**Fig 5:**
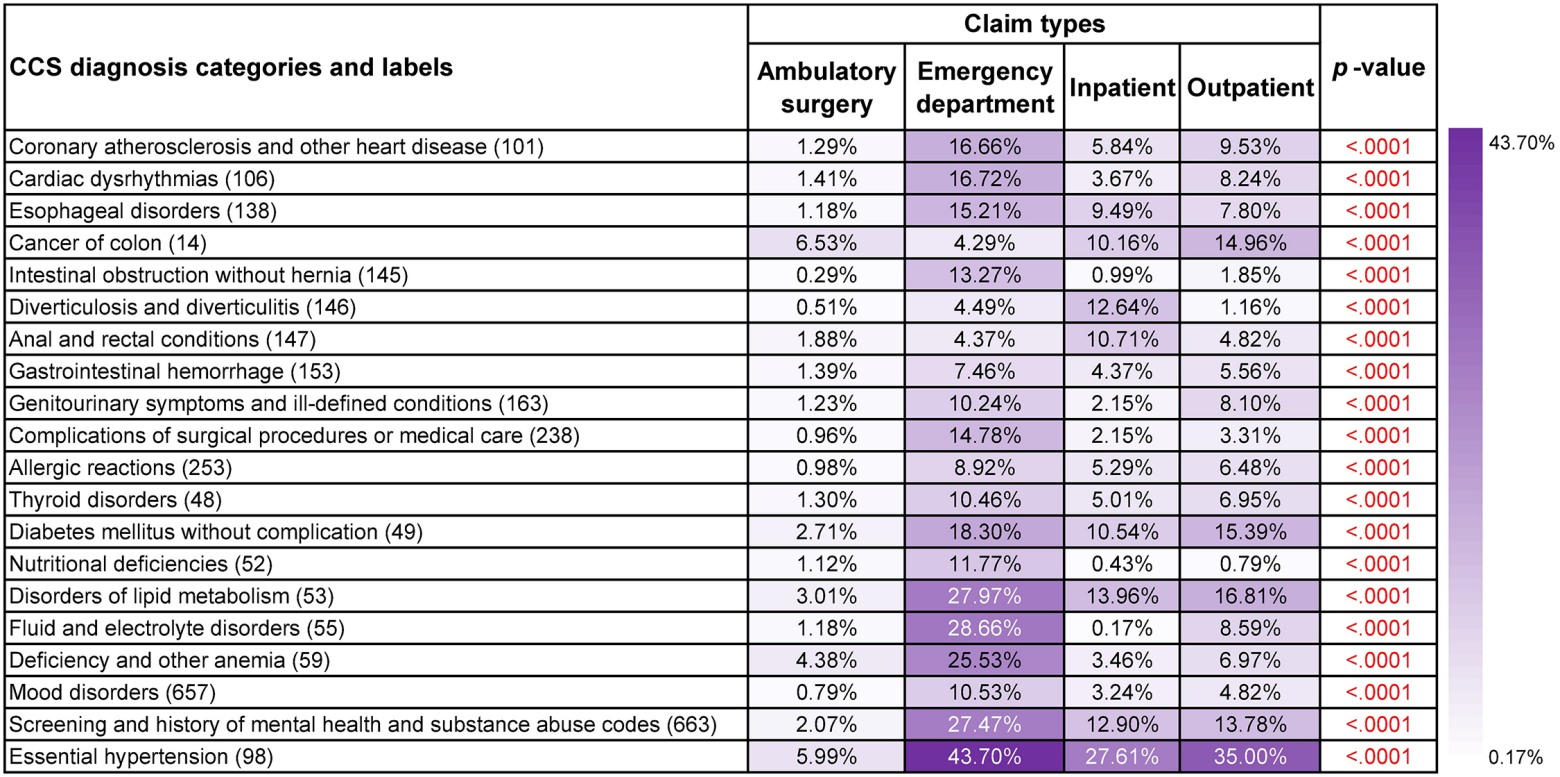
Supports and *p*-values of the most frequent diagnoses that co-occurred with rectum and anus cancer (15) in discharge records for different claim types. Age, gender, race, ethnicity and claim type were treated as covariates and *p*-values based on *F*-tests were used to assess their overall significance.

Distribution of frequent diagnosis co-occurrence patterns regarding claim types differed among all seven types of cancer (*p*-values<0.0001). For instance, colon cancer (14) were the most frequent diagnosis only in ambulatory surgery visits from patients having rectum and anus cancer (15) (Fig 5), biliary tract disease (149) were comparatively frequent in ambulatory surgery visits and inpatient hospital stays with respect to pancreas cancer (17) (S3 Table). However, some common patterns can still be identified. Most of the discharge records of lung and bronchus cancer (19) (S4 Table), prostate cancer (29) (S6 Table) and Non-Hodgkin’s lymphoma (38) (S7 Table) came from ambulatory surgery visits, least of them were from inpatient care. Most discharge records for rectum and anus cancer (15) (Fig 5) and liver and intrahepatic bile duct cancer (16) (S2 Table) were collected from emergency department visits.

#### Frequent co-occurrences of cancer diagnoses and procedure codes

**Fig 6:**
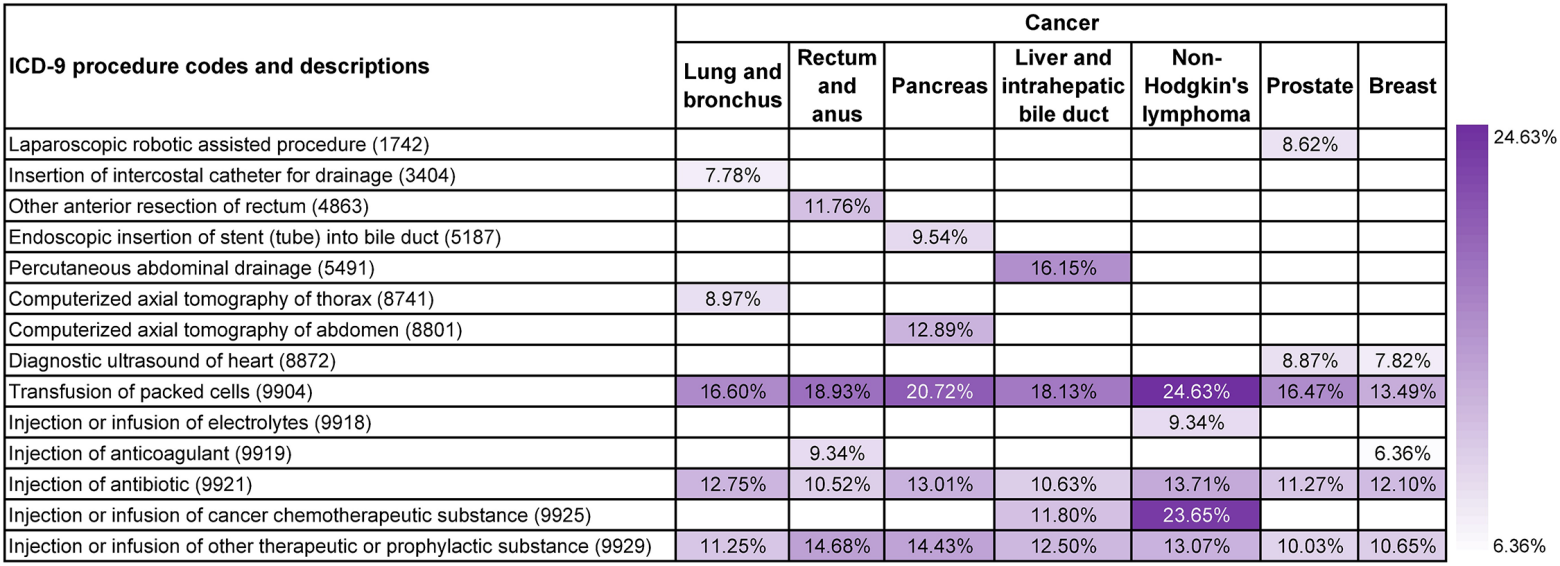
Supports of the most frequent procedures used to treat different types of cancer.

Fig 6 illustrates the procedure codes that co-occurred most frequently with different cancer diagnoses. We used discharge records that contained both targeted cancer diagnoses and valid procedure codes in this study. We also selected the top 20 most frequent procedure codes for each type of cancer, while only present top five most frequent procedure codes here. Transfusion of packed cells (9904), injection of antibiotic (9921) and injection or infusion of other therapeutic or prophylactic substance (9929) appeared in the top five most frequent procedure codes of all seven types of cancer and were usually in the first three places. Moreover, transfusion of packed cells (9904) always ranked the first for each type of cancer. Pattern disparities among different types of cancer could also be identified. Insertion of intercostal catheter for drainage (3404) (7.78%) and computerized axial tomography of thorax (8741) (8.97%) were popular regarding lung and bronchus cancer (19). Other anterior resection of rectum (4863) (11.76%) was more common in treating rectum and anus cancer (15). Computerized axial tomography of abdomen (8801) (12.89%) and endoscopic insertion of stent (tube) into bile duct (5187) (9.54%) were more frequent in results for pancreas cancer (17). Percutaneous abdominal drainage (5491) (16.15%) was popular in treating liver and intrahepatic bile duct cancer (16). Injection or infusion of electrolytes (9918) (9.34%) was more frequently used to treat Non-Hodgkin’s lymphoma (38). Laparoscopic robotic assisted procedure (1742) (8.62%) was only found in top five frequent patterns for prostate cancer (29). Injection or infusion of cancer chemotherapeutic substance (9925) was most frequently used to treat liver and intrahepatic bile duct cancer (16) (11.80%) and Non-Hodgkin’s lymphoma (38) (23.65%).

### Diagnosis sequence patterns

**Fig 7:**
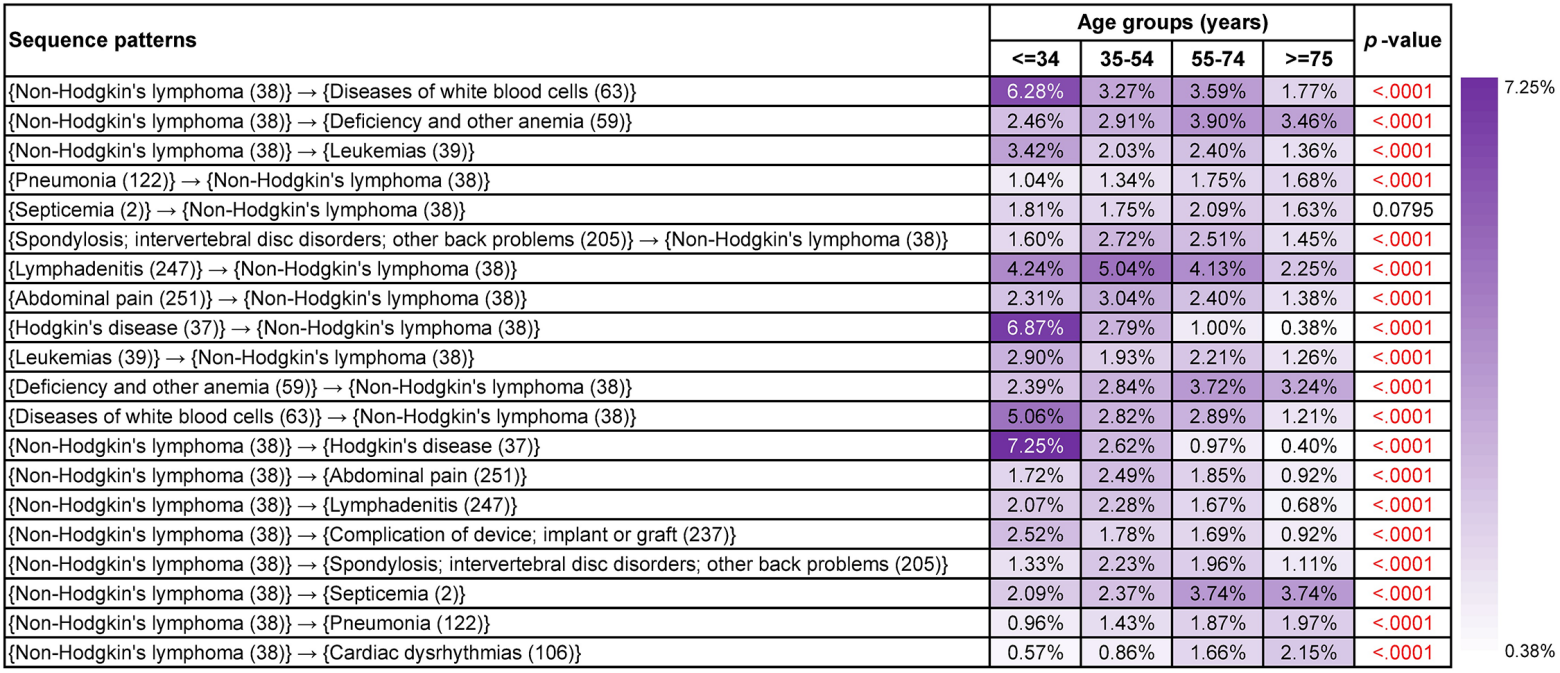
Supports and *p*-values of the most frequent sequence patterns found from patients who had Non-Hodgkin’s lymphoma (38) in different age groups. Age, gender, race and ethnicity were treated as covariates and *p*-values based on *F*-tests were used to assess their overall significance.

In our analyses, we searched on full patient diagnosis sequences using primary CCS categories only, because primary diagnoses could help detect more clinically meaningful patterns [31]. We also used both primary and secondary diagnoses and ran analyses on sequences from patients who had Non-Hodgkin’s lymphoma (38). Results are available in S7 Table. By comparing results presented in Figs 3 and 7 and S7 Table, we found that although a combination of primary and secondary CCS categories contained richer diagnosis information, the information could be redundant and noisy.

We only present length-2 diagnosis sequence patterns in this section, as longer sequences in our analyses usually consisted of repeated CCS categories representing follow-up visits rather than disease progression. Moreover, since only primary diagnoses were used in mining sequence patterns and patients having targeted diagnoses usually did not have these CCS categories as the primary diagnoses, supports of patterns presented in this section are comparatively lower than patterns generated using both primary and secondary diagnosis information (S7 Table).

#### Frequent sequence patterns in different age groups

Diagnoses in sequence patterns were usually more closely correlated with targeted cancers than those in co-occurrence patterns.

Non-Hodgkin’s lymphoma (38) was a typical cancer type where significant disparities with respect to age were observed. Fig 7 presents results of frequent sequence patterns for Non-Hodgkin’s lymphoma (38) in different age groups. Hodgkin’s disease (37), leukemias (39), diseases of white blood cells (63) and lymphadenitis (247) were four essential comorbidities. “{Non-Hodgkin’s lymphoma (38)}→{diseases of white blood cells (63)}” (<= 34 years old: 6.28%, *p*-value<0.0001), “{Hodgkin’s disease (37)}→{Non-Hodgkin’s lymphoma (38)}” (<= 34 years old: 6.87%, *p*-value<0.0001), “{Non-Hodgkin’s lymphoma (38)}→{Hodgkin’s disease (37)}” (<= 34 years old: 7.25%, *p*-value<0.0001), “{diseases of white blood cells (63)}→{Non-Hodgkin’s lymphoma (38)}” (<= 34 years old: 5.06%, *p*-value<0.0001) were four sequence patterns with the highest supports discovered from patients who were or under 34 years old. Besides, “{lymphadenitis (247)}→{Non-Hodgkin’s lymphoma (38)}” (35-54 years old: 5.04%, 55-74 years old: 4.13%, *p*-value<0.0001) was the most frequent sequence pattern among patients between 35-74 years old.

Patients who had rectum and anus cancer (15) (S1 Table) were more frequently diagnosed with colon cancer (14). Sequence patterns containing colon cancer (14) were the most common ones across all age groups (*p*-values<0.0001). Patients between 0-54 years old had pattern “{cancer of colon (14)}→{cancer of rectum and anus (15)}” (<= 34 years old: 7.68%, 35-54 years old: 11.94%) with supports slightly lower than its reversed sequence “{cancer of rectum and anus (15)}→{cancer of colon (14)}” (<= 34 years old: 8.56%, 35-54 years old: 12.27%). However, the picture was different among patients who were or over 55 years old: the former (55-74 years old: 10.93%, >= 75 years old: 6.66%) was more frequent than the latter (55-74 years old: 10.90%, >= 75 years old: 6.28%). Also, patients within this age group (>= 55 years old) were more likely to had pattern “{cancer of rectum and anus (15)}→{septicemia (2)}” (55-74 years old: 2.52%, >= 75 years old: 2.36%). Sequences containnig anal and rectal conditions (147) were more common among patients who were or under 54 years old (*p*-values<0.0001).

Patterns regarding liver and intrahepatic bile duct cancer (16) (S2 Table) also varied among different age groups. Hepatitis (6) and abdominal pain (251) were two significant diagnoses in those patterns. Sequence “{cancer of liver and intrahepatic bile duct (16)}→{hepatitis (6)}” (35-54 yeas old: 5.75%, 55-74 years old: 5.73% years old, *p*-value<0.0001) and sequence “{hepatitis (6)}→{cancer of liver and intrahepatic bile duct (16)}” (35-54 yeas old: 6.95%, 55-74 years old: 6.14% years old, *p*-value<0.0001) were more common among patients who were 35-74 years old. Pattern “{cancer of liver and intrahepatic bile duct (16)}→{abdominal pain (251)}” (35-54 years old: 4.95%, *p*-value<0.0001) and “{abdominal pain (251)}→{cancer of liver and intrahepatic bile duct (16)}” (35-54 years old: 7.07%, *p*-value<0.0001) were more frequent among patients between 35-54 years old.

#### Frequent sequence patterns in different gender groups

**Fig 8:**
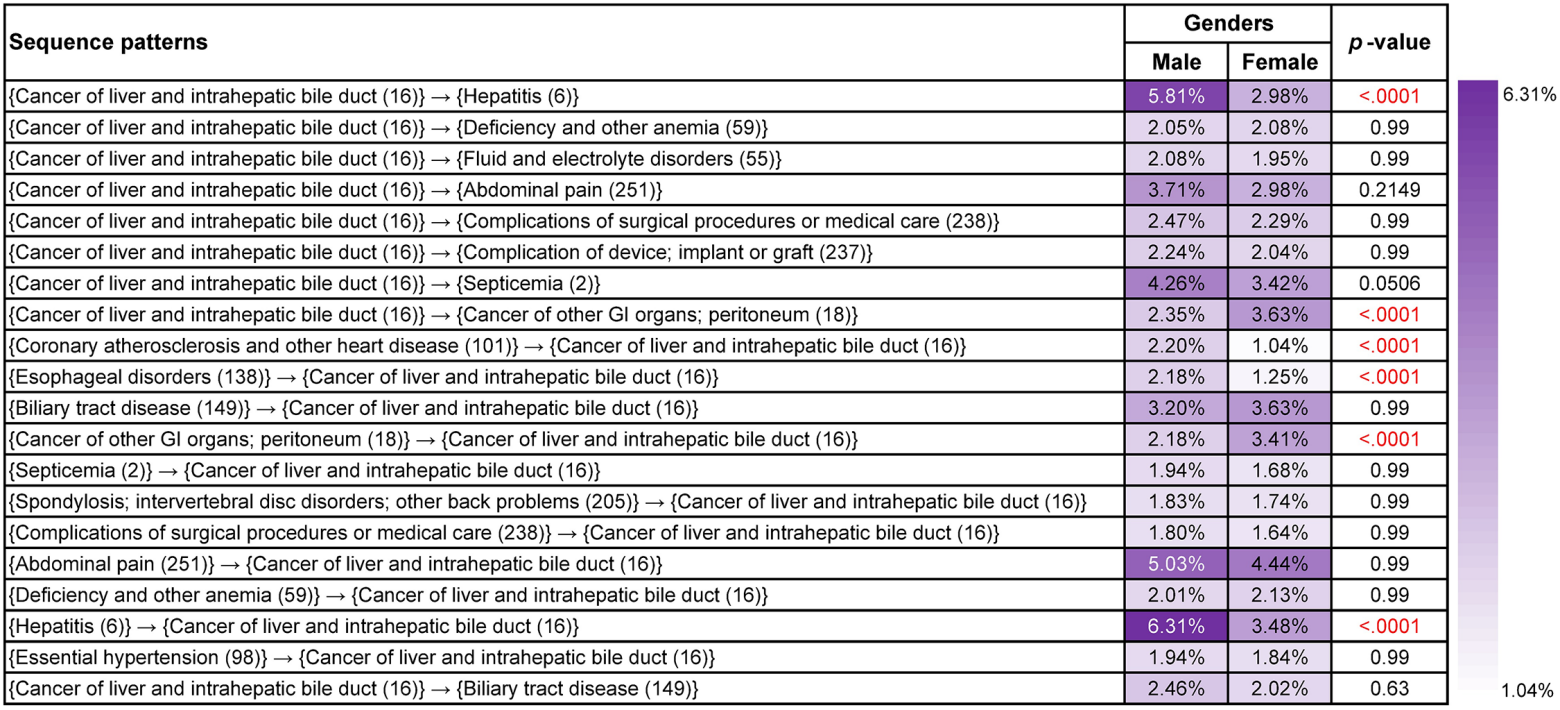
Supports and *p*-values of the most frequent sequence patterns found from patients who had liver and intrahepatic bile duct cancer (16) in different gender groups. Age, gender, race and ethnicity were treated as covariates and *p*-values based on *F*-tests were used to assess their overall significance.

Similar to disease co-occurrence patterns, distribution of top frequent sequence patterns for cancer types that are less gender specific, such as rectum and anus (15) (S1 Table), pancreas cancer (17) (S3 Table), lung and bronchus cancer (19) (S4 Table) and Non-Hodgkin’s lymphoma (38) (S7 Table) demonstrated similar trends for both genders, but supports for males were usually higher than females. However, liver and intrahepatic bile duct cancer (16) was an exception. As shown in Fig 8, female patients were more likely to have cancer of other GI organs or peritoneum (18). These patterns were “{cancer of liver and intrahepatic bile duct (16)}→{cancer of other GI organs or peritoneum (18)}” (male: 2.35%, female: 3.63%, *p*-value<0.0001), “{cancer of other GI organs or peritoneum (18)}→{cancer of liver and intrahepatic bile duct (16)}” (male: 2.18%, female: 3.41%, *p*-value<0.0001). Although breast cancer (24) occured more commonly in females than in males, the trends of frequent sequence patterns were similar between different gender groups (S5 Table). For instance, “{nonmalignant breast conditions (167)}→{cancer of breast (24)}” (male: 3.86%, female: 8.00%, *p*-value<0.0001) and “{cancer of breast (24)}→{nonmalignant breast conditions (167)}” (male: 1.58%, female: 5.23%, *p*-value<0.0001) were highly popular with both genders.

## Discussion

Although our work focused on discoveries of patterns and disparities in different patient groups, there were many common diagnoses appearing in almost results of all seven types of cancer, especially in diagnosis co-occurrence patterns. In co-occurrence patterns, patients who were and over 75 years old had much higher risk having cardiovascular diseases such as coronary atherosclerosis (101) and cardiac dysrhythmias (106). Essential hypertension (98) was another popular diagnosis with elder patients and it was high on the list of the top 20 frequent co-occurrence patterns of every cancer. Besides, disorders of lipid metabolism (53), fluid and electrolyte disorders (55), diabetes mellitus without complications (49) and deficiency and other anemia (59) were also common diagnoses that co-occurred frequently with different cancer diagnoses.

In our study, we used both primary and secondary diagnoses to discover disease co-occurrence patterns and only primary diagnoses to identify sequence patterns. As aforementioned, primary diagnosis codes are the main reason for a hospital visit and secondary diagnosis codes represent conditions that co-exist during the same hospital visit or stay. The sequence patterns usually contained diagnoses that were highly correlated with each targeted cancer, but many of these diagnoses were not available in the most frequent co-occurrence patterns. Thus, primary diagnosis information was more accurate and precise, and secondary diagnosis codes provided richer but redundant and noisy information. Moreover, sequence patterns conveyed information that were time dependent. For example, the support of a sequence was usually different from the support of its reversed sequence. This phenomenon might weigh significantly in studying disease progression.

## Conclusions

Open data initiatives make large scale healthcare data available and provide us a unique opportunity for discovering patterns using data mining methods. We adopted Apriori algorithm and cSPADE algorithm to discover frequent disease co-occurrence and sequence patterns among cancer patients in New York State using SPARCS data. We studied seven types of cancer with high incident rates in New York State and focused on disparities of diagnosis co-occurrence patterns and diagnosis sequence patterns from patients’ diagnosis histories with respect to age, gender as well as claim types. Our results suggest that the methods can generate potentially interesting and clinically meaningful disease co-occurrence and sequence patterns, which can be used to study comorbidities and disease progression for improving the management of multiple diseases of cancer patients.

## Supporting information

S1 Table Other results for rectum and anus cancer (15). (XLSX)

S2 Table Other results for liver and intrahepatic bile duct cancer (16). (XLSX)

S3 Table Other results for pancreas cancer (17). (XLSX)

S4 Table Other results for lung and bronchus cancer (19). (XLSX)

S5 Table Other results for breast cancer (24). (XLSX)

S6 Table Other results for prostate cancer (29). (XLSX)

S7 Table Other results for Non-Hodgkin’s lymphoma (38). (XLSX)
